# Computational and Experimental Models of Cancer Cell Response to Fluid Shear Stress

**DOI:** 10.3389/fonc.2013.00044

**Published:** 2013-03-05

**Authors:** Michael J. Mitchell, Michael R. King

**Affiliations:** ^1^Department of Biomedical Engineering, Cornell UniversityIthaca, NY, USA

**Keywords:** cancer metastasis, circulating tumor cells, mechanotransduction, shear stress, blood, interstitial flow

## Abstract

It has become evident that mechanical forces play a key role in cancer metastasis, a complex series of steps that is responsible for the majority of cancer-related deaths. One such force is fluid shear stress, exerted on circulating tumor cells by blood flow in the vascular microenvironment, and also on tumor cells exposed to slow interstitial flows in the tumor microenvironment. Computational and experimental models have the potential to elucidate metastatic behavior of cells exposed to such forces. Here, we review the fluid-generated forces that tumor cells are exposed to in the vascular and tumor microenvironments, and discuss recent computational and experimental models that have revealed mechanotransduction phenomena that may play a role in the metastatic process.

## Introduction

To initiate the metastatic spread of cancer through the bloodstream, tumor cells must transit through microenvironments of dramatically varying physical forces. Cancer cells must be able to migrate through the stroma, intravasate through the endothelium into blood or lymphatic vessels, flow within the vessels and subsequently extravasate through the endothelium, and migrate and colonize in tissue at a secondary site (Chambers et al., 2002; Steeg, 2006; Chaffer and Weinberg, 2011). In soft tissues, cancer cells are exposed to mechanical forces due to fluid shear stress, hydrostatic pressure, and tension and compression forces (Butcher et al., 2009; DuFort et al., 2011). During intravasation and extravasation, cells undergo dramatic elastic deformations to transmigrate through endothelial cell–cell junctions (Tseng et al., 2004; Wirtz et al., 2011). Once in the circulation, tumor cells must be able to withstand immunological stress, blood cell collisions, and hemodynamic shear forces, while also utilizing flow to adhere to the endothelial wall and subsequently extravasate to form a secondary tumor (Hughes and King, 2011). Across all of these steps, a deeper understanding is needed of how biophysical forces contribute to biochemical changes in cancer cells, which can reveal novel strategies in the treatment of metastasis.

Fluid shear stress is one of the prominent forces that cells are exposed to, and its effects on blood cells, endothelial cells, smooth muscle cells (SMCs), and others have been extensively studied (Moazzam et al., 1997; Civelek et al., 2002; Li et al., 2005). However, much less is known about fluid shear stress effects on tumor cells. Cancer cells experience two main types of fluid shear stress: stresses generated by blood flow in the vascular microenvironment, and those generated by interstitial flows in the tumor microenvironment (Michor et al., 2011; Swartz and Lund, 2012). Stresses generated by interstitial and blood flows could contribute to the metastatic process by enhancing tumor cell invasion and circulating tumor cell (CTC) adhesion to blood vessels, respectively. However, it is difficult to predict tumor cell behavior to such forces; it is difficult to experimentally measure such flows in the tumor microenvironment (Shieh and Swartz, 2011), and there is a general lack of data on force-dependent CTC receptor–ligand interactions with the endothelium (Cheung et al., 2011). Sophisticated experimental techniques coupled with computational modeling are needed to predict cell behavior upon exposure to varying complex physical forces.

In this review, we provide examples of both experimental and computational methods to model and predict how cancer cells respond to fluid shear forces. We begin by describing the fluid shear forces that cancer cells are exposed to in both the tumor and vascular microenvironments, generated mainly by blood and interstitial flows. An overview is provided on computational modeling to estimate the forces exerted on cells in blood and tissues, along with simulations to predict cell behavior under such flows. We then describe recent cancer cell mechanotransduction phenomena upon exposure to fluid shear stress, such as altering cancer cell resistance to fluid shear stress, sensitivity to apoptosis-inducing ligands, and invasive and migratory potential. We conclude with current computational models that aim to integrate fluid shear forces with chemical signaling, such as the effect of the glycocalyx on transmitting physical forces and inducing mechanotransduction in cancer cells, as well as the integration of signal transduction networks into adhesive dynamics (AD) simulations to predict cell adhesion in the microvasculature.

## Fluid Shear Stress Exposure in the Tumor Microenvironment

Cancer cells in the tumor microenvironment are exposed to multiple physical forces including fluid shear stress, hydrostatic pressure, tension, and compression, which have been treated in detail previously (Butcher et al., 2009; Wirtz et al., 2011; Swartz and Lund, 2012). Here, cancer cell exposure to physical forces generated by interstitial flows will be discussed briefly.

**Figure 1:**
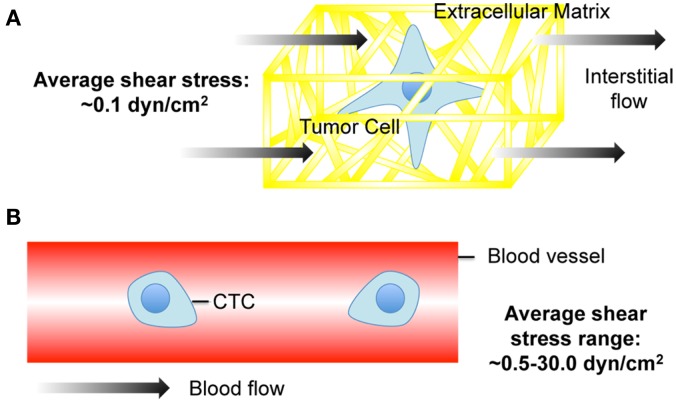
**Cancer cell exposure to the tumor and vascular microenvironments**. **(A)** Tumor cell exposed to interstitial flow in a collagen matrix (Swartz and Fleury, 2007). **(B)** Circulating tumor cell (CTC) exposed to fluid shear forces in a blood vessel.

Interstitial flow is the slow movement of fluid around cells and through the pores of the extracellular matrix (ECM) that comprise the interstitium (Figure 1A). One of the main functions of interstitial flow is lymphatic drainage, which returns plasma from leaky capillaries back to the bloodstream. Drainage occurs due to Starling’s forces, which are osmotic and hydrostatic pressure gradients between blood vessels, interstitium, and the lymphatics (Schmid-Schonbein, 1990). The composition of interstitial fluid can vary depending on the location in the body, but in soft tissues is generally similar to the blood plasma that leaks from capillaries, and contains approximately 40% of the protein concentration of plasma (Swartz and Fleury, 2007). The velocities of interstitial flows are believed to range from 0.1 to 1.0 μm s^−1^ in normal tissues (Chary and Jain, 1989; Dafni et al., 2002). Cell surface shear stresses are believed to be on the order of 0.1 dyn cm^−2^ (Pedersen et al., 2007; Tarbell and Shi, 2012).

Interstitial flows can be elevated significantly in the tumor microenvironment, and play a crucial role in tumor progression. Chary and Jain (1989) utilized fluorescence recovery after photobleaching (FRAP) to measure interstitial fluid velocities of bovine serum albumin in normal and neoplastic tissues. Harrell et al. utilized live imaging of tumor-bearing mice to measure downstream lymph flow via injection of fluorescent nanoparticles. Measurements were performed in both normal and neoplastic tissues; all tumor-bearing mice in the study showed increases in lymph flow, compared to control mice without tumors (Harrell et al., 2007).

Elevated interstitial flows in the tumor microenvironment are likely due to increased tumor interstitial fluid pressure (IFP). Boucher and Jain (1992) implanted colon adenocarcinoma cells into mice, tracked the development of the tumor vasculature using intravital microscopy, and measured IFP using micropipettes and a servo-null system. IFP measurements increased significantly as the vasculature developed, demonstrating that tumor interstitial hypertension is associated with tumor angiogenesis (Boucher et al., 1996). IFP is elevated in a uniform manner throughout tumors, and drops significantly at the tumor periphery (Boucher et al., 1990). Thus, IFP gradients facilitate fluid flow outward from tumors, presenting a mass transport barrier for the delivery of chemotherapeutics (Netti et al., 1995; Lunt et al., 2008).

Increased IFP also effects tumor biology, as it applies increased physical force to the ECM and alters interstitial flows that the tumor and surrounding cells are exposed to. Nearby lymphatic vessels respond to elevated interstitial flow by upregulating chemokine CCL21 expression, along with cell adhesion molecules E-selectin and ICAM-1 (Miteva et al., 2010). Secretion of CCL21 directs tumor cells toward lymphatic vessels (Shields et al., 2006), while ICAM-1 and E-selectin upregulation enhances cell transmigration into lymphatic vessels (Johnson et al., 2006; Miteva et al., 2010). Lymph nodes can also be affected, as increased interstitial flows aid in lymph node architecture remodeling to colonize tumor cells, as well as protect the tumor from an immune response (Shieh and Swartz, 2011).

Fibroblasts, which deposit, turn over, and remodel ECM to maintain connective tissue homeostasis, can aid in tumor progression due to elevated interstitial flows. Elevated interstitial flows can upregulate transforming growth factor beta-1 (TGF-β_1_) expression (Ng et al., 2005; Ng and Swartz, 2006; Wipff et al., 2007; Ahamed et al., 2008), which can induce a tumor-associated fibroblast phenotype characterized by enhanced contractility and increased secretion of cytokines, angiogenic growth factors, and matrix metalloproteinase (MMPs) (Hinz et al., 2002; De Wever et al., 2004a,b; Orimo and Weinberg, 2006). Recently, Shieh et al. (2011) demonstrated that interstitial flows can enhance tumor cell invasion when cocultured with dermal fibroblasts in a 3D collagen matrix. Fibroblast invasion was enhanced due to increased expression of TGF-β (Chaffer and Weinberg, 2011) and MMPs, while it appeared that tumor cell invasion was enhanced due to fibroblast-dependent remodeling of the ECM (Shieh et al., 2011).

## Fluid Shear Stress Exposure in Vascular Microenvironment

To enter the vascular microenvironment, cancer cells penetrate surrounding tissue and enter nearby blood and lymphatic vessels in a process called intravasation. The underlying mechanisms that govern intravasation are not well understood; it is still in question whether intravasation is an active or passive process (Bockhorn et al., 2007), and whether tumor cells enter the circulation via endothelial cell-cell junctions or directly through endothelial cells themselves (Khuon et al., 2010). Regardless of their mechanism of entry, cancer cells are exposed to a new set of conditions once in the vascular microenvironment, including immunological stress, collisions with blood cells, and hemodynamic shear forces, all of which can affect their survival and proliferation.

Cancer cells are primarily exposed to erythrocytes, leukocytes, and platelets upon entering the bloodstream, as studies have shown that the concentration of cancer cells in the blood of patients is on the order of one in a million leukocytes (Maheswaran and Haber, 2010), or one in a billion blood cells (Yu et al., 2011). Exposure to such cells can lead to immunological stresses and blood cell collisions that can affect cancer cell viability (Wirtz et al., 2011), although there is evidence that the association of platelets with cancer cells in the bloodstream can promote tumor metastasis (McCarty et al., 2000; Gay and Felding-Habermann, 2011).

Cancer cells are also exposed to hemodynamic shear forces in the bloodstream (Figure 1B), which range from 0.5 to 4.0 dyn cm^−2^ in the venous circulation and 4.0–30.0 dyn cm^−2^ in arterial circulation (Turitto, 1982). Shear rates can range from approximately 160 s^−1^ in veins to 900 s^−1^ in arteries. Such shear stresses and rates can affect cancer cell viability and thus the chances of metastasis. For example, B16 melanoma cell exposure to fluid shear stress in a cone-and-plate viscometer at shear rates greater than 300 s^−1^ induced a significant loss of cell viability (Brooks, 1984).

In contrast, fluid shear stress is an essential component of cancer metastasis, as it is critical for cancer cell adhesion to the endothelial cell wall and subsequent extravasation into tissues. A variety of cancer cell lines are known to express sialylated carbohydrate ligands, which adhesively interact with selectin proteins on the inflamed microvasculature (Gout et al., 2008; Köhler et al., 2010; Läubli and Borsig, 2010). Thus, cancer cells are believed to undergo an adhesion cascade similar to leukocytes, which consists of a sequence of steps involving tethering, rolling, and firm adhesion to the endothelium (Chambers et al., 1995; Coussens and Werb, 2002). Multiple studies have documented that a variety of tumor cell lines bind to E-selectin proteins under physiological shear stresses of the post-capillary venules (Giavazzi et al., 1993; Barthel et al., 2009).

Much less is known about fluid shear stresses that cancer cells could be exposed to in lymphatic vessels. Lymphatic vessels have been stained with fluorescein isothiocyanate (FITC)-labeled macromolecules to measure lymphatic flow in single lymphatic capillaries of humans *in vivo* using intravital capillary microscopy (Fischer et al., 1996). The recorded median linear velocity in lymphatic capillaries was 9.7 μm s^−1^, and shear stresses in lymph node sinuses have been estimated to be 10-fold lower than hematogenous shear stresses (Resto et al., 2008). Despite the dramatic decrease in shear stress levels, parallel plate flow chamber studies have shown that human head and neck squamous carcinoma cells can bind to lymphocyte L-selectin at lymphatic shear stress levels of 0.07–0.08 dyn cm^−2^ (Resto et al., 2008).

## Computational Methods to Model Cell Exposure to Interstitial Flows

**Figure 2:**
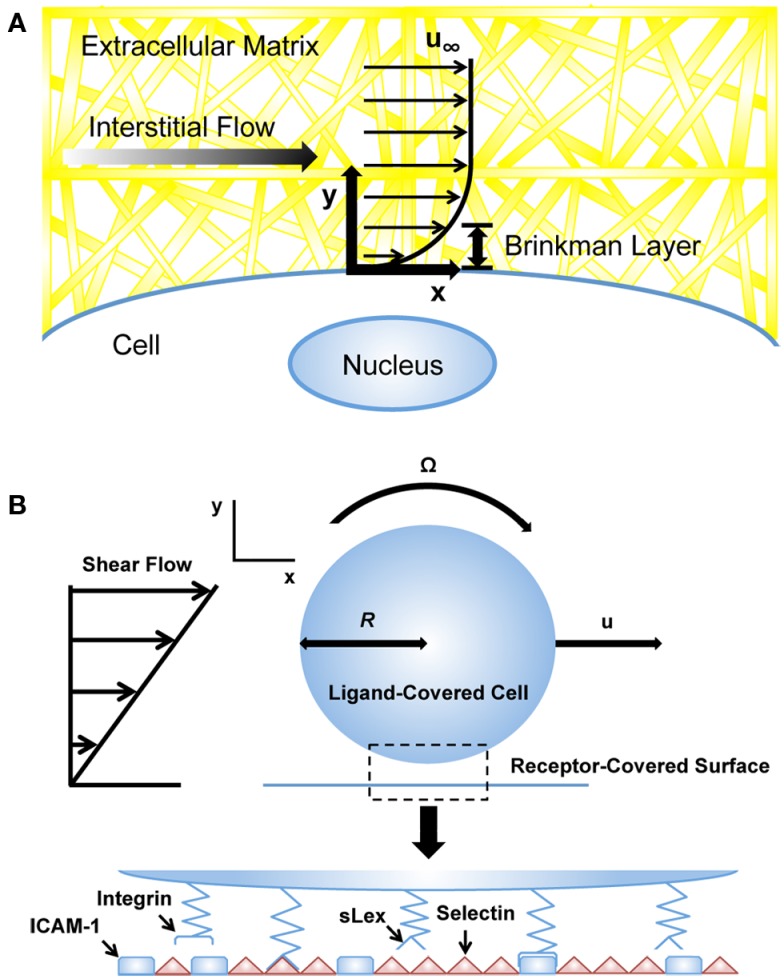
**Computational models of cells exposed to blood and interstitial flows**. **(A)** Computational models utilizing the Brinkman equation to estimate interstitial flow-generated shear stresses on the cell surface (Tarbell and Shi, 2012). *u*_∞_: velocity far from cell surface. **(B)** Adhesive dynamics (AD) simulations to predict selectin-mediated adhesion to the endothelium (Bhatia et al., 2003). *u*, velocity; *R*, cell radius; sLex, sialyl-Lewis-x; ICAM-1, intercellular adhesion molecule-1.

Interstitial flow mechanics were initially described by French hydraulics engineer Henry Darcy, who studied the flow of water through sand beds as a means of providing filtered drinking water to his city. During his studies, he developed the formula known as Darcy’s law:

$$ū=\frac{-K\nabla P}{\mu },$$

where *K* is the permeability of the medium, ∇*P* is the pressure gradient vector, μ is the viscosity of the fluid, and ū is the averaged velocity through the bulk. Darcy’s law works well when the average velocity or mass flow rate needs to be determined, but is first order with respect to velocity. To account for interstitial flows between boundaries, Brinkman developed a second order term, taking into account no-slip boundary conditions adjacent to bounding walls (Figure 2A; Brinkman, 1949). The Brinkman equation is described as:

$$\nabla P=-\frac{\mu }{K}ū+\mu \nabla^{2}ū.$$

Permeability measurements have been performed for a variety of tissues *in vitro*, *in vivo*, and *ex vivo*, including muscle (Rasheid Zakaria et al., 1997), dermis (Bert and Reed, 1995), cartilage (Levick, 1987), tumors (Netti et al., 2000; McGuire et al., 2006), and fibrin and collagen gels (Diamond, 1999; Ng and Swartz, 2003), making the Darcy and Brinkman equations useful for both experimental measurements of interstitial flows and computational models of cells exposed to such flows.

Initial models of interstitial flows exerted on cells were developed for tissues including smooth muscle, cartilage, and bone (Kwan et al., 1984; Grodzinsky et al., 2000; Hellmich and Ulm, 2005). For example, Wang and Tarbell (1995) modeled the tunica media of an artery as a periodic array of cylindrical, impermeable SMCs embedded in a matrix consisting of collagen and proteoglycans, and used Brinkman’s theory to model interstitial flow across the tissue. The model was able to estimate the effective hydraulic permeability of the tissue and shear stresses exerted on SMCs, which were estimated to be on the order of 1.0 dyn cm^−2^ despite exposure to low interstitial flows (Wang and Tarbell, 1995). In an early model describing the mechanics of interstitial-lymphatic transport, Swartz et al. developed a theoretical and experimental model demonstrating how interstitial flow is dependent on hydraulic conductivity, elasticity, and lymphatic conductance. They then utilized this model to examine fluid balance in normal and chronically swollen (edematous conditions) mouse tails, in which they found that remodeling of the matrix dampened and eventually stagnated fluid movement in the case of edema (Swartz et al., 1999).

## Computational Methods to Model Cell Behavior in the Circulation

A variety of computational methods have been developed to model cell behavior in the vascular microenvironment, including adhesive dynamics (AD), which has been utilized to simulate cell adhesion to the endothelial cell surface under flow (Hammer and Lauffenburger, 1987; Hammer and Apte, 1992). The motivation of such simulations is to predict how adhesiveness quantitatively depends on factors such as shear rate and viscosity, which can reveal adhesion phenomena that might not necessarily follow intuition. AD is a mechanically rigorous cell adhesion simulation that models individual molecular bonds as compliant springs. In the simulation, the cell can be modeled as a rigid spherical particle covered with a random distribution of adhesion molecules (Figure 2B). The endothelial cell wall can be modeled as a surface covered with counter-receptor molecules of random distribution. Bonds randomly form between adhesion molecules of the cell and counter-receptors on the wall; these bonds can then break contingent on the appropriate kinetics, which depend on the instantaneous force loading on the spring endpoints. The rates of bond formation and rupture can be calculated using the Bell model for kinetics of single biomolecular bond failure (Bell, 1978; Bell et al., 1984):

$$k_{r}=k_{r}^{0}\exp\left(\frac{r_{0}F}{k_{b}T}\right)$$

where *k_r_* is the rate of dissociation, $k_{r}^{0}$ is the unstressed off-rate, *F* is the force on the bond, *r*_0_ is the reactive compliance, *T* is the temperature, and *k*_b_ is the Boltzmann constant. The rate of bond formation follows from the Boltzmann distribution of affinity, while also incorporating the effects of relative motion between the cell and surface (King et al., 2005). To solve the algorithm, unbound receptors in the defined contact area are first tested for formation against the probability:

$$P_{f}=1-\exp\left(-k_{f}\Delta t\right)$$

where *P_f_* is the probability of bond formation, and *t* is time. Next, bound receptors are tested for breakage against the probability:

$$P_{r}=1-\exp\left(-k_{r}\Delta t\right)$$

where *P_r_* is the probability of bond rupture. External forces and torques on the cell are then summed, and a mobility calculation determines the motion of the cell. Cell and bond positions are updated based on the kinematics of cell motion. Torques exerted by fluid flow and hydrodynamic forces cause the adherent cell to slowly roll forward on a reactive surface. The motion of fluid is governed by the Stokes equation:

$$\mu \nabla^{2}u=\nabla P,\;\nabla \cdot u=0,$$

where *u* is the velocity, μ is the viscosity of the fluid, and *P* is the pressure. No-slip boundary conditions are applied at the cell surface and the planar wall.

While AD has not yet been used to model cancer cell adhesion, many simulations have been performed using leukocytes, which can be a close parallel to a CTC that has undergone the epithelial-mesenchymal transition (EMT). Chang et al. (2000) utilized AD to develop a state diagram for leukocyte adhesion under flow. In the diagram, observed adhesive behaviors (rolling, firm adhesion, or no adhesion) were plotted at given dissociation rates and bond interaction lengths, which spanned several orders of magnitude. Caputo and Hammer (2005) incorporated deformable microvilli with clustered adhesion molecules onto the surface of the simulated leukocyte, and found that the deformability of the microvilli can affect the cell’s ability to roll on a surface. King and Hammer (2001a,b) modeled the effect of cell–cell hydrodynamic interactions on the dynamics of leukocyte adhesion using Multiparticle AD (MAD), which revealed a mechanism for secondary hydrodynamic recruitment of leukocytes to the blood vessel wall, independent of leukocyte–leukocyte contact interactions.

Critical parameters of AD simulations are the kinetics of selectin–carbohydrate bonds, as force-dependent dissociation rates dictate the rolling adhesion of leukocytes. Numerous studies have investigated the kinetics for leukocyte selectin ligands using experimental techniques such as flow chamber tethering experiments, atomic force microscopy, and dynamic force spectroscopy (Smith et al., 1999). However, such kinetics for newly identified selectin ligands expressed by metastatic tumor cells, which appear distinct from those found on the surface of leukocytes (Thomas et al., 2008; Shirure et al., 2012), have not yet been well characterized. Future experimental studies measuring bond dissociation kinetics for selectins and CTC selectin ligands will enable the development of more predictive computational models of cancer cell adhesion to microvasculature.

## Current Experimental Models of Cancer Cell Mechanotransduction

### Fluid shear stress alters cancer cell response to apoptosis-inducing ligands

The targeting and treatment of CTCs within the circulation is currently being investigated as an approach to prevent their metastatic spread. For example, microfluidic devices coated with E-selectin conjugated liposomal doxorubicin have been shown to capture cancer cells from flow, deliver doxorubicin into the cell, and induce cell death (Mitchell et al., 2012a,b). Similarly, microfluidic devices immobilized with E-selectin and tumor necrosis factor (TNF)-related apoptosis-inducing ligand (TRAIL) have been shown to capture and kill cancer cells (Rana et al., 2009) while exerting minimal toxic effects on human leukocytes (Rana et al., 2012). However, little is known about how fluid shear stress exposure can affect cancer cell response to drug treatments.

**Figure 3:**
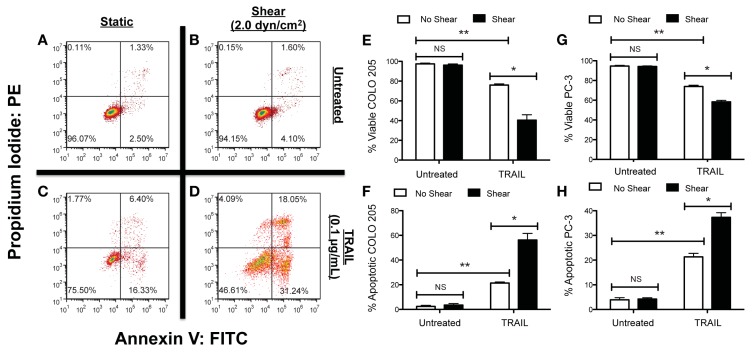
**Fluid shear stress sensitizes cancer cells to the apoptosis-inducing ligand TRAIL**. Colorectal adenocarcinoma COLO 205 cells exposed to non-shear conditions **(A)** and fluid shear stress **(B)**, respectively. COLO 205 cells treated with TRAIL and then exposed to non-shear conditions **(C)** and fluid shear stress **(D)**. Lower left-hand and right-hand quadrants of each flow cytometry figure represent viable cells and cells in early stages of apoptosis, respectively. Upper left-hand and right-hand quadrants represent cells undergoing necrosis and late stage apoptosis, respectively. Percentage of viable **(E)** and apoptotic **(F)** COLO 205 cells after treatment with TRAIL followed by exposure to non-shear or shear conditions (*n* = 3). Percentage of viable **(G)** and apoptotic **(H)** PC-3 cells treated under the same conditions (*n* = 3). PE, phycoerythrin; FITC, fluorescein isothiocyanate. Error bars represent 95% confidence intervals. *P < 0.05. **P < 0.01. NS, non-significant. Figure reprinted with permission from Mitchell and King (2012).

Our recent study examined how colorectal adenocarcinoma COLO 205 and prostate adenocarcinoma PC-3 cancer cell exposure to physiologically relevant fluid shear stresses in a cone-and-plate viscometer altered their response to TRAIL (Figure 3; Mitchell and King, 2013). Experiments were devised in such a way that fluid shear stress alone had negligible effects on cancer cell death. Cancer cells were treated with both TRAIL, which can bind to death receptors DR4 and DR5 on the cancer cell surface to initiate apoptosis (Ashkenazi, 2002), and doxorubicin, which induces cell death via inhibition of topoisomerase II and DNA intercalation (Young et al., 1981; Osheroff et al., 1994). Interestingly, treatment of both COLO 205 and PC-3 cancer cell lines with TRAIL followed by exposure to 2.0 dyn cm^−2^ of fluid shear stress significantly increased the number of apoptotic cells, compared to TRAIL-treated cancer cells exposed to static conditions. The sensitization effect was both fluid shear stress dose- and time-dependent, as the number of apoptotic cells increased over a range of shear stress magnitudes (0.05–2.0 dyn cm^−2^) and shear stress exposure times (1–120 min). However, such sensitization was not evident in doxorubicin treatment, as the percentage of apoptotic cells remained nearly identical in doxorubicin-treated samples exposed to either fluid shear stress or static conditions. The results indicated that such sensitization could be receptor-mediated apoptosis specific.

It is possible that death receptors on the cancer cell surface can sense and respond to fluid shear forces. The idea of circulating cells expressing mechanosensitive receptors has recently been investigated in leukocytes (Makino et al., 2006; Mitchell and King, 2012), where it is believed that G-protein coupled receptors can sense fluid shear stress and alter neutrophil adhesion to the microvasculature. However, little is known about the effects of fluid shear stress on CTC surface receptors. Insight into the mechanistic basis of such processes could reveal new strategies for treating cancer cells in the circulation, and reducing the likelihood of metastasis.

### Cancer cell resistance to fluid shear stress

**Figure 4:**
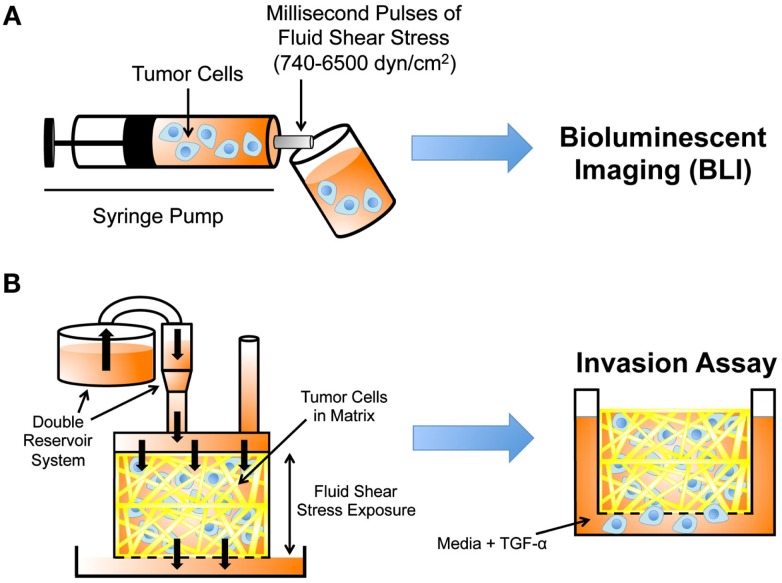
**Experimental techniques to study cancer cell mechanotransduction**. **(A)** Microfluidic protocol to deliver millisecond pulses of fluid shear stress to tumor cells (Barnes et al., 2012). Tumor cell resistance to fluid shear stress determined using bioluminescent imaging (BLI). **(B)** Darcy flow apparatus for the application of fluid shear stress in 3D to tumor cells embedded in collagen (Qazi et al., 2011). Shear stress-exposed cells are then placed in a modified Boyden chamber to measure their migratory and invasive potential in the presence of TGF-α.

Recently, a microfluidic protocol was developed to assess cancer cell resistance to fluid shear stress (Barnes et al., 2012). In the protocol, dilute cancer cell suspensions were drawn up into a syringe, which was then loaded into an automatic syringe pump (Figure 4A). Cancer cell suspensions were exposed to brief, millisecond pulses of high fluid shear stress as they were expelled from the syringe pump, and subsequently analyzed for cell viability using bioluminescent imaging. The maximum fluid shear stress that cancer cells were briefly exposed to in this experiment reached 6400 dyn cm^−2^. Note that CTCs are momentarily exposed to shear stresses as high as 3000 dyn cm^−2^ at vessel bifurcations, in the heart, and near the walls of large blood vessels (Strony et al., 1993; Malek et al., 1999). While cancer cell viability decreased after repeated millisecond pulse exposures to high fluid shear stress, the study revealed that cancer cells of epithelial origin were surprisingly resistant to fluid shear stress, in comparison to non-transformed epithelial cells. Resistance to fluid shear stress was dependent on several oncogenes, as *myc-* and *ras-*transformed cell lines showed an increase in fluid shear stress resistance. The resistance response required extracellular calcium and actin polymerization, as the absence of calcium or treatment with EGTA, cytochalasin D, or ROCK inhibitor Y27632 all reduced cancer cell viability upon fluid shear stress exposure. In particular, extracellular calcium is important for cellular repair mechanisms based on an extracellular calcium-dependent membrane resealing process (Terasaki et al., 1997).

### Fluid shear stress regulates cancer cell invasive potential

Prior work has shown that the chemokine gradients generated by interstitial flows can enhance tumor cell migration (Shields et al., 2007), however it is not well understood whether fluid shear stress can regulate intrinsic properties of cancer cells, thus altering their invasive potential. Recent work by Qazi et al. (2011) detailed a Darcy flow apparatus for the application of fluid shear stress to a 3D collagen gel embedded with glioma cells, coupled with a modified Boyden chamber invasion assay. In the apparatus, a double reservoir system applied hydrostatic pressure, which drove media throughout the 3D collagen gel and exerted shear stress on the glioma cells (Figure 4B). Cancer cells were exposed to fluid shear stresses ranging from 0.1 to 0.6 dyn cm^−2^. The media filtrate from the gel was collected in a separate reservoir, and the media collected was used to calculate flow rates, velocities, and shear stresses. Collagen gels were removed at the end of the flow period, and placed within modified Boyden chambers containing TGF-α to initiate invasion assays.

Fluid shear stress significantly reduced U87 and CNS-1 glioma cell migration by as much as 92% and 58% respectively, when compared to controls. Migration suppression was not due to flow-induced chemokine gradients, however, as cells were exposed to fluid shear stress followed by exposure to TGF-α in static Boyden chambers. Invasion was dependent on matrix metalloproteinases (MMPs), as MMP-1 and MMP-2 gene expression was significantly downregulated in cancer cells upon exposure to 0.55 dyn cm^−2^ fluid shear stress. Previous studies have shown that fluid shear stress can affect MMP expression and activity in non-tumor cell types such as fibroblasts, chondrocytes, and SMCs (Yokota et al., 2003; Garanich et al., 2007; Shi and Tarbell, 2011), however this was one of first studies revealing that fluid shear stress-induced mechanotransduction is involved in interstitial flow-induced cancer cell motility.

### Interstitial flow induces tumor cell focal adhesion kinase activation

A recent study investigated two competing mechanisms which can alter tumor cell migration upon exposure to interstitial flow: an autologous chemotaxis-based mechanism which distributes autocrine chemokine via convection to create a chemokine gradient, and a mechanism whereby interstitial flow activates focal adhesion kinase (FAK) and modulates forces critical for tumor cell migration (Fincham and Frame, 1998; Sieg et al., 1998). Polacheck et al. (2011) developed a microfluidic cell culture system to investigate the effects of interstitial flow on the directional bias and dynamics of tumor cell migration in a 3D matrix. Utilizing two channels separated by a region in which tumor cells were suspended in a 3D collagen gel, a pressure gradient was applied across the gel to generate consistent interstitial flow velocities ranging from 0.3 to 3.0 μm s^−1^, representative of a range of values measured *in vivo* (Dafni et al., 2002; Heldin et al., 2004). Confocal reflective microscopy was used to track cell migration under flow, and it was found that interstitial flow and cell seeding density can both influence the direction of tumor cell migration.

Upon exposure to interstitial flow at low seeding densities, MDA-MB-321 metastatic breast cancer cells migrated in the downstream direction, or “with the flow.” However, cancer cells exposed to interstitial flow at high seeding densities migrated upstream, or “against the flow.” Treatment with CCR7 blocking antibodies, to block the binding of secreted ligand CCL21 needed to initiate autologous chemotaxis, caused cells to shift their migration directionality and migrated upstream upon exposure to flow. Cells that migrated in the opposite direction of flow displayed increased phosphorylation at Tyr-397 in FAK, which plays a role in Src kinase activation and focal adhesion formation (Li et al., 1997; Jalali et al., 1998). Upon blockage of Src kinase activity, upstream tumor cell migration decreased and displayed random cell migration.

## Current Advances in Modeling Mechanotransduction Phenomena

### Modeling glycocalyx effects on interstitial fluid shear stress transmission to cancer cells

The glycocalyx is a layer of proteoglycans and glycoproteins that covers eukaryotic cells, which can serve as a mechanosensor of fluid shear stress in endothelial cells and SMCs (Yao et al., 2007; Shi et al., 2011). Tumor cells also possess a glycocalyx (Krähling et al., 2009), however its effects as a mechansensor have not been previously investigated. It has been hypothesized that fluid shear stress generated by interstitial flows is too weak to induce mechanotransduction.

**Figure 5:**
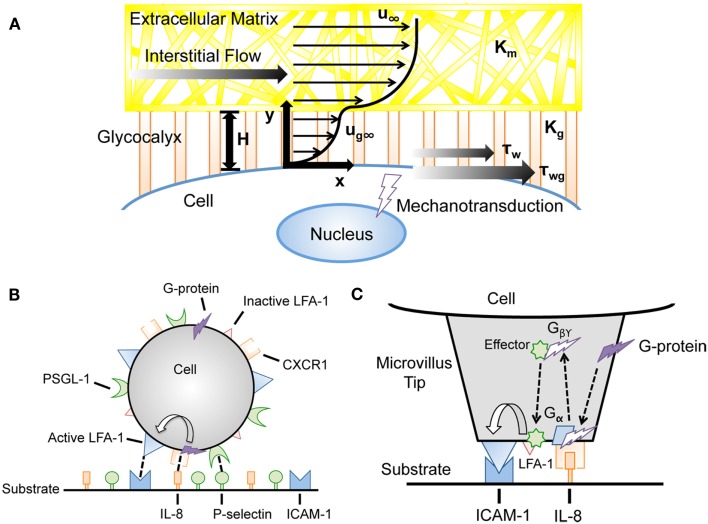
**Advances in computational modeling reveal mechanotransduction phenomena**. **(A)** Interstitial flow models incorporating the force-transducing cell glycocalyx to determine interstitial flow contributions to fluid shear stress-dependent mechanotransduction (Tarbell and Shi, 2012). *u*_∞_, velocity far from cell surface; *K*_m_, matrix Darcy permeability; *K*_g_, glycocalyx Darcy permeability; *H*, glycocalyx layer thickness; *u*_g∞_, velocity profile in glycocalyx; τ_w_, surface fluid stress; τ_wg_, surface solid stress. **(B,C)** Incorporation of cell signaling networks to predict flow-mediated cell adhesion in the presence of chemoattractants (Caputo and Hammer, 2009). IL-8, interleukin-8; PSGL-1, P-selectin glycoprotein ligand-1; LFA-1, lymphocyte function-associated antigen-1.

Tarbell and Shi (2012) recently developed a computational model to estimate the interstitial flow-generated fluid and solid stresses on the surface of a glycocalyx-covered cell embedded in ECM (Figure 5A). Previously estimated parameters such as the Darcy permeability of the ECM, tumor cell glycocalyx thickness, and interstitial fluid flow velocity were incorporated into the model to calculate the fluid and solid stresses on the cell surface. Brinkman equations were used to describe interstitial fluid flow through pores of both the ECM and glycocalyx. A previously described model (Secomb et al., 2001) was used to calculate mechanical equilibrium of forces in the direction of flow to calculate the solid stresses transmitted via the glycocalyx. While fluid stresses exerted on the tumor cell surface were estimated to be quite low (less than 0.1 dyn cm^−2^), the solid stresses transmitted to the cell via the glycocalyx were predicted to be over 5.0 dyn cm^−2^, a magnitude which is known to activate endothelial cells (Malek et al., 1999). Future models could incorporate mechanical effects along with chemical signaling pathways to better predict cancer cell mechanotransduction in tissues.

### Integrating signal transduction networks into adhesive dynamics simulations

Recently, signal transduction models were incorporated into AD simulations to couple signaling pathways with cell adhesion. In the model, leukocytes were assigned a random spatial distribution of integrin lymphocyte function-associated antigen-1 (LFA-1), in addition to selectin ligands such as PSGL-1. The reactive surfaces were covered with selectin molecules and intracellular adhesion molecule-1 (ICAM-1), which binds to active LFA-1 and mediates firm arrest. Krasik et al. (2006) integrated the mitogen-activated protein kinase (MAPK) signal transduction pathway as a modular Hill function within the AD framework to model neutrophil arrest with deterministic activation. Selectin ligation triggered the MAPK cascade in this model, which can cause inactive LFA-1 to become activated, enabling binding to ICAM-1 and subsequent neutrophil arrest. This model has since incorporated a stochastic signal transduction model, utilizing a Monte Carlo simulation within the microvilli of model neutrophils (Krasik et al., 2008).

Caputo et al. generated an AD simulation with an integrated signal transduction network that incorporates selectin, integrin, and chemokine interactions between the neutrophil and the substrate. A random distribution of the G-protein coupled receptor CXCR1 and chemokine interleukin-8 (IL-8) were displayed on the leukocyte and the reactive surfaces, respectively (Figure 5B,C). CXCR1 can interact with IL-8, which initiates a signaling cascade leading to LFA-1 activation on the cell (Caputo and Hammer, 2009). Beste et al. (2012) developed a model of T-lymphocyte arrest by combining AD with a kinetic model for chemokine-triggered inside-out integrin activation. The model incorporated signaling data measured in experiments to simulate the time scale for T-lymphocyte arrest, and provided a predictive simulation for understanding chemokine control of T-lymphocyte recruitment. The integration of signal transduction networks into AD simulations could prove particularly useful for the study of cancer metastasis, as molecular defects could be implemented within the signaling cascade to predict its effects on CTC adhesion to the endothelium.

### Computational models of integrin–ligand interactions at the cell-ECM interface

A model based on the AD simulation was developed to both chemically and mechanically model integrin dynamics at the cell-ECM interface (Paszek et al., 2009). Paszek et al. developed the model to determine whether the cell glycocalyx and the chemical and physical parameters of the ECM can control the formation of integrin clusters, which act as mechanical anchors and can regulate cell survival, motility, differentiation, and morphogenesis (Hynes, 2002; Miranti and Brugge, 2002; Berrier and Yamada, 2007). Integrin–ligand bonds were modeled as individual Hookean springs, and the Bell model was utilized to calculate kinetic rates of bond formation and rupture, which are distance-dependent (Bell, 1978; Bell et al., 1984). In addition, the model included a lattice spring model (LSM) of the cell–ECM interface, consisting of a lattice of interconnecting nodes and springs to calculate the stress–strain behavior of the interface (Ostoja-Starzewski et al., 1996). Model parameters including the glycocalyx, membrane, and bond spring constants, on- and off-rates, and receptor and ligand density were estimated based on experimental measurements.

Integrin clustering began as a fast process, as simulations showed that new integrin bond formation events were more likely to occur near existing integrin bonds where the separation distance between integrins and ligands was reduced. However, bond rearrangements due to bond breakage and reformation were found to slow down the integrin clustering process over time. Glycocalyx thickness also affected integrin clusters, with larger, denser clusters forming with increased glycocalyx thickness. The interplay between integrin–ligand affinity and cell–ECM repulsion due to the glycocalyx also affected clustering; high affinity interactions coupled with thinner glycocalyx resulted in bound integrin receptors with minimal clustering. A thicker glycocalyx relative to integrin bond length, along with an adequate receptor–ligand affinity, resulted in both integrin binding and clustering. Integrin clustering increased due to increases in the ratio of glycocalyx stiffness to membrane stiffness, as it increased the minimal matrix ligand density. Integrin clustering was shown to be sensitive to ECM stiffness; compliant substrates could not promote cooperative binding, while integrin clustering increased with increasing substrate stiffness above 2000 Pa. While the computational model only incorporates basic biology, a combination of the mechanical model with molecular interactions revealed cell adhesion behavior observed in experiments (Cluzel et al., 2005; Paszek et al., 2009). Future models should focus on the incorporation of applied fluid shear forces, along with integrin–cytoskeleton interactions, to predict how adhesions on the cancer cell surface can sense and respond to the tumor microenvironment.

## Conclusion

Fluid shear stresses generated by blood and interstitial flows alter cancer cell behavior in the vascular and tumor microenvironments, respectively, and contribute to the progression of cancer metastasis. Interstitial flow-generated forces elevate tumor IFP, and create challenges to chemotherapeutic delivery to the tumor interior. Such forces also induce phenotypic changes of cells in the surrounding microenvironment, which enhance tumor cell migration and invasion. Shear flows in the circulation affect tumor cell viability while also playing a role in CTC adhesion to the endothelium, a crucial step for subsequent tumor cell extravasation and metastasis. Recent experimental studies have revealed that fluid shear stress can modulate intrinsic characteristics of cells, in addition to the extrinsic roles of fluid flow that have been previously documented. Cancer cell mechanotransduction observed in recent experiments, including tumor cell resistance to shear stress, regulation of migration and invasion, and sensitivity to chemotherapeutics, have potentially wide ranging implications for metastasis. Recent computational models have incorporated mechanical fluid forces with chemical signaling networks, along with mechanotransducing components on the cancer cell surface, such as the glycocalyx. Future approaches utilizing computational models of fluid shear stress effects on intrinsic tumor cell signaling networks, coupled with *in vitro* and *in vivo* experimental validation, may better predict cell behavior in such dynamic microenvironments, and potentially provide novel approaches for the prevention of metastasis.

## Conflict of Interest Statement

The authors declare that the research was conducted in the absence of any commercial or financial relationships that could be construed as a potential conflict of interest.
